# A Critical Appraisal of the Diagnostic and Prognostic Utility of the Anti-Inflammatory Marker IL-37 in a Clinical Setting: A Case Study of Patients with Diabetes Type 2

**DOI:** 10.3390/ijerph20043695

**Published:** 2023-02-19

**Authors:** Zvonimir Bosnić, František Babič, Viera Anderková, Mario Štefanić, Thomas Wittlinger, Ljiljana Trtica Majnarić

**Affiliations:** 1 Department of Family Medicine, Faculty of Medicine, Josip Juraj Strossmayer University of Osijek, Huttlerova 4, 31000 Osijek, Croatia; 2 Department of Cybernetics and Artificial Intelligence, Faculty of Electrical Engineering and Informatics, Technical University of Košice, 06601 Košice, Slovakia; 3 Department of Nuclear Medicine, Faculty of Medicine, Josip Juraj Strossmayer University of Osijek, Huttlerova 4, 31000 Osijek, Croatia; 4 Department of Cardiology, Asklepios Hospital, University of Göttingen, 38642 Goslar, Germany

**Keywords:** type 2 diabetes, older adults, chronic inflammation, cytokine IL-37, diagnostic utility

## Abstract

Background: The role of the cytokine interleukin-37 (IL-37) has been recognized in reversing inflammation-mediated metabolic costs. The aim was to evaluate the clinical utility of this cytokine as a diagnostic and prognostic marker in patients with type 2 diabetes (T2D). Methods: We included 170 older (median: 66 years) individuals with T2D (females: 95) and classified as primary care attenders to assess the association of factors that describe patients with plasma IL-37 levels (expressed as quartiles) using multinomial regression models. We determined the diagnostic ability of IL-37 cut-offs to identify diabetes-related complications or patient subgroups by using Receiver Operating Characteristic analysis (c-statistics). Results: Frailty status was shown to have a suppressive effect on IL-37 circulating levels and a major modifying effect on associations of metabolic and inflammatory factors with IL-37, including the effects of treatments. Situations in which IL-37 reached a clinically significant discriminating ability included the model of IL-37 and C-Reactive Protein in differentiating among diabetic patients with low–normal/high BMI ((<25/≥25 kg/m^2^), and the model of IL-37 and Thyroid Stimulating Hormone in discriminating between women with/without metabolic syndrome. Conclusions: The study has revealed limitations in using classical approaches in determining the diagnostic and prognostic utility of the cytokine IL-37 in patients with T2D and lain a foundation for new methodology approaches.

## 1. Introduction

Interleukin-37 (IL-37) is a member of the IL-1 cytokine family, otherwise known for its role in inflammation promotion [1]. This cytokine, formerly known as family member 7 of the IL-1 cytokine family, was characterized by computational cloning as having a role as a negative regulator of IL-18 which, in synergism with IL-1β, acts as the critical proinflammatory cytokine of this cytokine family [2]. IL-37 is a part of the mechanisms of self-control developed during evolution to limit the harmful effects of excessive inflammation [3,4]. The broad suppressor activities of IL-37 in both innate and adaptive immunity arise from its dual modes of action, which include the effects induced by the binding of this cytokine to the cell surface IL-18 decoy receptors and the effects that are exerted by its binding to the nuclear DNA (Deoxyribonucleic acid), where it impedes the transcription of proinflammatory genes [5,6]. In addition, complex mechanisms are involved in its activation since IL-37 transcripts are released in the cytosol in the precursor forms, which may account for additional variability in this cytokine’s abundance and specific actions [7,8]. Besides its anti-inflammatory activity, IL-37 acts to restore cell metabolic homeostasis during inflammation and reverse chronic inflammation’s metabolic costs [9,10].

The results of experimental studies cannot be directly translated into real-life situations. In human diseases, in different conditions that occur under the same disease label, the prevailing proinflammatory or anti-inflammatory effect of IL-37 may relate to either IL-37 genetic variations or other specific patient-related or disease-related contexts. Regarding IL-37 genetic variations, differences in the expression of several existing IL-37 isoforms, either as mRNA (messenger Ribonucleic acid) variants or variations in single nucleotide polymorphisms, were found to influence the abundance of the circulating IL-37 and disease pathways [11,12,13,14,15,16]. However, the level of knowledge of the clinical consequences of these variations is still low [7,8,17,18]. It is worth noting that cytokine regulation, and in particular regarding IL-37, as a negative regulator of inflammation is generally more complex in multifactorial age-related conditions (such as type 2 diabetes (T2D), where patients with the same diagnostic label share multiple pathophysiology pathways and exhibit heterogeneous clinical expression), than in autoimmune and immunologically mediated diseases, which are characterized by high-impact genetic and environmental exposures, and which are more homogeneous in phenotypic expression [19,20]. That differences in IL-37 gene polymorphisms in combination with behavioral or other specific patient-related factors may differently direct phenotypic variability was demonstrated in a recent epidemiologic study, where authors searched for IL-37 gene polymorphisms in individuals with and without hypercholesterolemia [21].

Due to its functioning at the crossroad of inflammatory and metabolic pathways, IL-37 has attracted growing interest in cardio-metabolic conditions for its translational perspective. The theoretical framework is based on the evidence indicating that obesity and its related disorders, metabolic syndrome (MS) (glucose-related metabolism impairment associated with abdominal obesity and hypertension), T2D, and cardiovascular disease (CVD) are those factors that contribute the most to inflammation associated with accelerated aging [22]. Increased activity of the innate immune system as well as increased production of metabolic intermediates and reactive oxygen species due to the glucose-related metabolism impairment generate inflammatory signals [23,24]. The proinflammatory microenvironment turns the balance from the predomination of anti-inflammatory regulatory T cells (Treg) to the development of the proinflammatory Th1/Th17 immune pathway by metabolic reprogramming and epigenetic alterations, for which IL-37 acts as a reversing factor [9,25,26]. The therapeutic potential of IL-37 has been demonstrated in experimental models [27,28,29]. However, clinical studies in this setting are insufficient to provide the specific biological properties of IL-37 across a range of clinical conditions [30,31,32].

Before the clinical utility of IL-37 becomes possible in cardio-metabolic conditions, there is a need to establish the reference range for this cytokine in healthy adults and patient subgroups [33]. The problem with these conditions is that they are characterized by high complexity, as already shown for patients with T2D. That means that multiple comorbidities, which change in scope and intensity can influence the pathophysiology pathways and inflammatory responses, depending on the dynamics of disease progression and patient age [34]. It implies the need to search for new research approaches when validating the prognostic value of circulating IL-37 in cardio-metabolic conditions [35]. Accordingly, this study aimed to identify the range values of plasma levels of IL-37 in older primary care (PC) patients diagnosed with T2D, to critically evaluate their diagnostic and prognostic relevance, and to identify limitations of using classical approaches in determining the diagnostic utility of this cytokine in older patients with T2D.

## 2. Materials and Methods

### 2.1. Participants and Study Design

Participants were patients diagnosed with T2D, aged 50 years or more (median 66), and attendees in a PC setting. The study was conducted in 2020 and lasted for four months. Four PC practices were included, and doctors agreed to participate in the study. The Expert and Ethics Council of the Health Centre, where these PC practices were located, approved the study (ID: 1433-1/020). Only patients who were able to come to the doctor personally, but not those dependent on the care of others, were recruited. They were selected consecutively at their visits, except for those who met the exclusion criteria. For the exclusion criteria, we excluded acute conditions, malignant diseases in the active treatment phase, noticeable or diagnosed cognitive impairments, and individuals with an amputated lower limb, transplanted kidneys, or those on chronic renal replacement therapy. The calculation of the sample size, based on the significance level of 0.05 and power of 0.8, indicated a minimum sample size of 180 individuals (G*Power, 3.1.9.4.). The final number of participants included in the study was 170 (Male: Female, 75:95), as we adjusted the sample size to the size of the cytokine diagnostic kits.

### 2.2. Data Collection

Participants were described by a total of 62 variables, including sociodemographic characteristics, anthropometric measures, comorbidities, medications, frailty, nutritional status, markers of inflammation, and laboratory tests indicating metabolic status and renal function (Table S1a,b—Supplementary Materials). Some data were taken from patient health records, such as comorbidities and medication prescription information. During their visits, patients undertook anthropometric measurements and an assessment of their frailty and nutritional status. They were referred to the county hospital’s biochemical laboratory for venipuncture and laboratory testing.

To assess frailty, we used Fried’s phenotype model, which requires a walking exercise and hand grip strength assessment by the dynamometer and involves information on the level of activity, weight loss, and subjective feeling of exhaustion [36]. Positive 1-2 criteria out of a maximum of five indicate pre-frailty, and three or more positive criteria indicate frailty. Nutritional status was assessed by using the 18-item Mini Nutritional Assessment—Short Form (MNA-SF) test [37]. This test can discriminate among good nutritional status, risk of malnutrition, and malnutrition. The level of sarcopenia (muscle loss) was determined by measuring mid-arm circumference (mac) [38]. Body mass index (BMI, kg/m^2^) was used to determine general obesity, and waist circumference (wc) was used to determine the abdominal type of obesity. According to the guidelines, for older patients with T2D and characterized with multimorbidity, the threshold of HbA1c (Glycated hemoglobin) of 8.0 mmol/l or even 9.0 mmol/L can be considered a reasonable hyperglycemia control [39]. To determine whether patients had MS, we used the modified criteria of the National Cholesterol Education Program, Adult Treatment Panel III (NCP ATP III) definition [40]. The NCP ATP III definition of MS is: ≥2 of the following: wc ≥ 102 cm (88 cm for F), Triglycerides (TG) ≥ 1.7, HDL (High Density Lipoprotein)-cholesterol < 1.0 (1.2 for F), and diagnosis of hypertension.

Among laboratory tests, we also performed Thyroid-Stimulating Hormone (TSH) screening to detect patients with latent hypothyroidism—the common condition in patients with T2D, which can contribute to metabolic disorders [41].

Renal function decline, a common disorder in patients with T2D due to the development of diabetic nephropathy, was assessed from serum creatinine levels and information on patient age and gender by using the Modification of Diet in Renal Disease (MDRD) equation and online calculator [42]. An estimated glomerular filtration rate (eGFR) ≤ 60 mL/min was considered to indicate decreased renal function, corresponding with stages 3 and 4 of chronic renal insufficiency [43]. Diagnoses of other major diabetic complications (such as CVD, including coronary artery disease (CAD), chronic heart disease (CHD), cerebrovascular disease, and periphery artery disease), as well as of diabetic retinopathy were recorded in patient health records if the specialist examinations confirmed these diagnoses. Since cardiac imaging is necessary to evaluate CHD, and this data was not systematically recorded in patient health records, information was missing on the grades of CHD progression [44]. For prescribed medications, we used data on the prescription rates of the main groups of antidiabetic and antihypertensive drugs, on the hypolipidemic drugs “statins”, and on non-steroidal anti-inflammatory drugs (NSAID), all of which may have influenced metabolic parameters or the level of inflammation [45,46].

As a marker of chronic inflammation, we used the neutrophil-to-lymphocyte ratio (NLR), which can be estimated from the complete differential blood count [47]. We also used classical markers of inflammation and nutritional status, C-reactive protein (CRP), number of erythrocytes, hemoglobin, and hematocrit [48,49]. For IL-37 analysis, a part of the blood sample was separated, placed into heparinized tubes, and centrifuged for plasma. These specimens were transported in the transporter refrigerator to the Laboratory for Clinical Immunology and Allergology Diagnostics of the University Hospital Centre of Osijek, the administrative center of the eastern part of Croatia. For the determination of IL-37, the quantitative sandwich immunoassay technique was used (IL-37 Human Uncoated ELISA kit, Invitrogen, ThermoFisher Scientific, SAD).

### 2.3. Statistical Analysis

The data description was provided as mean ± SD (standard deviation) or the median and interquartile range (IQR) for numerical data and as absolute and relative frequencies for categorical data. Discrimination among patients by levels of IL-37 was expressed as quartiles of IL-37. The collinearity and multicollinearity were investigated for numerical attributes using correlation analysis (Spearman’s correlation coefficient) and Variance Inflation Factor (VIF). A value of VIF between 1 and 5 indicates a moderate correlation between the given predictor variable and other predictor variables in the model, but this is often not severe enough to require attention. A value greater than 5 indicates a potentially severe correlation between the given predictor variable and other predictor variables in the model. In this case, the coefficient estimates and p-values in the regression outputs are likely unreliable. The methods used to assess differences in other examined variables among quartiles of IL-37 were the Chi-square (χ^2^) test or the Fisher’s exact test for categorical variables, and the one-way analysis of variance (ANOVA) or the Kruskal–Wallis rank–sum test for numerical variables, depending on the type of distribution (standard or not). The Games-–Howell post-hoc test was used to compare differences between the quartile pairs for numerical variables that showed significant differences. The significance level of *p* < 0.05 was considered statistically significant in all cases. Associations of variables with quartiles of IL-37 were assessed by using the multinomial logistic regression (MLR) model from R statistics.

The Akaike Information Criterion (AIC) measured the model’s predictive performance quality. To estimate the optimal cut-off values of IL-37 and other inflammatory markers for patient subgroups, we used the function “cutpointr” from R statistics. This function works in a way to take the sum of sensitivity and specificity to maximize the metric (separation) function. The Receiver Operating Characteristics Curve (ROC) was used to test the predictive power of the identified cut-off values [50]. We used the ROC and the Area Under Curve (AUC) for evaluation, also known as the c-statistics [51]. The AUC metric varies between 0.5 and 1.00 (an excellent value). A value of AUC above 0.80 indicates a good classifier.

## 3. Results

**Figure 1:**
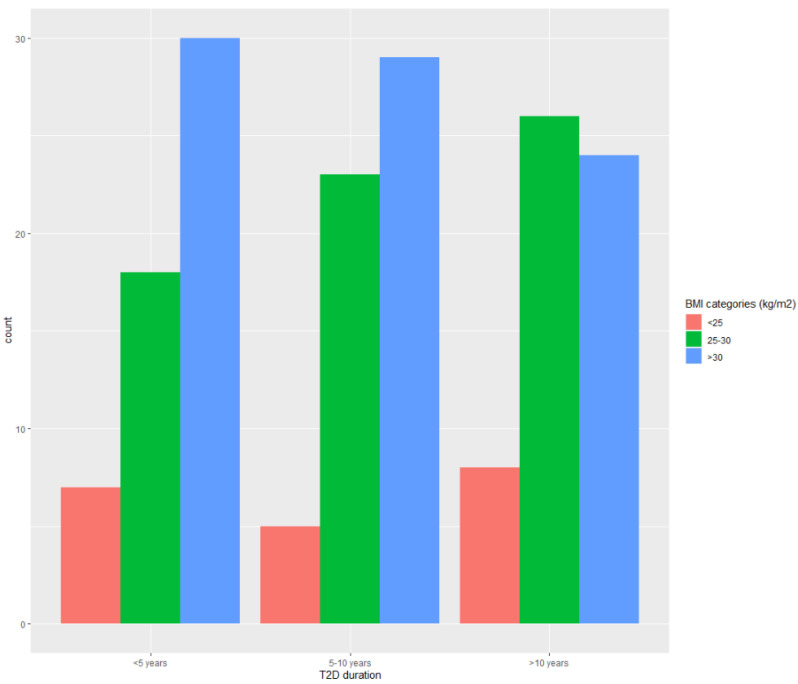
Distribution of patients according to T2D duration and BMI categories.

Participants were mostly 50–75 years old, and women participated more than men (Table S1a,b—Supplementary Materials). They were primarily overweight/obese (Figure 1). Most of the patients also had MS, as confirmed by the fact that a large proportion of them expressed the abdominal (visceral) type of obesity (wc ≥ 88 cm for females and ≥102 for males), and the majority were diagnosed with hypertension. There were patients with short-term (0–5 years) and long-term (>10 years) hypertension and T2D duration. Most of the patients were in a good nutritional state, and only a small proportion were at risk of malnutrition. No individuals had severe sarcopenia, as indicated by the fact that no one had mac ≤ 22 cm. Only about a fifth of patients were frail (31/170). In most patients, HbA1c was <8.5%, indicating well-controlled hyperglycemia.

Concerning diabetic complications, about one-third of patients were diagnosed with CAD. There was a high proportion of those diagnosed with CHD, although information on severity grades was missing. About two-thirds of participants had decreased renal function but, in most cases, renal function decline was of a mild (eGFR 90–60 mL/min) or a moderate degree (eGFR < 60–45 mL/min). When compared to the epidemiologic data, a high proportion of patients had diabetic retinopathy (>30%) [46]. Of non-cardiovascular comorbidities, the descriptive data indicated a high burden of musculoskeletal diseases and anxiety disorders. A high proportion of patients had been prescribed antihypertensive drugs of the ACE-INH/ARB (angiotensin-converting enzyme inhibitors/angiotensin receptor blockers) group and hypolipidemic statin drugs, indicating that family physicians in the study area have made efforts to adhere to the guidelines [39]. In addition, they were more prone to prescribe the old-fashioned antidiabetic drug, metformin, as the first-line therapy, while newly recommended cardio- and renal-protective drugs, GLP1ra (Glucagon-like peptide-1 receptor agonists) and SGLT2inh (Sodium-Glucose Transport Protein 2 inhibitors), were prescribed at low rates [39,46].

**Figure 2:**
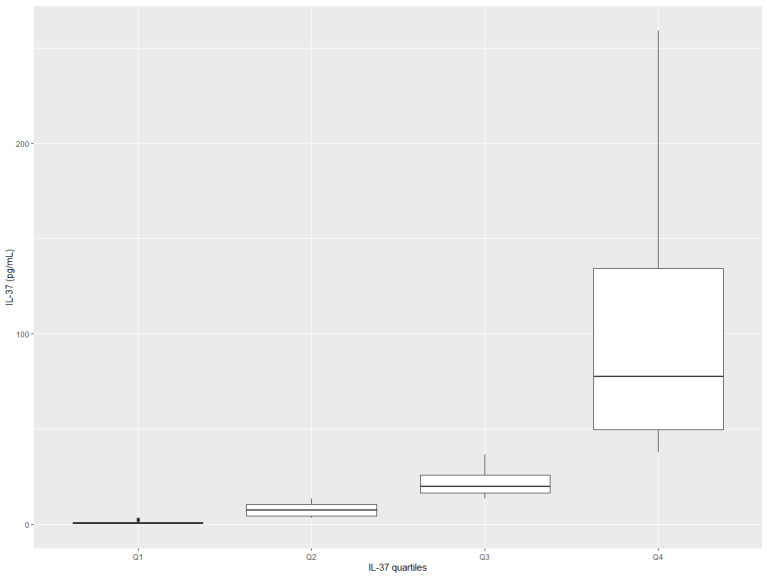
Distribution of IL-37 among participants (box-plot diagram).

The values of IL-37 for most of the patients oscillated between 3.40 and 38.0 pg/mL, being skewed around the median value (Table 1 and Figure 2). The variability of IL-37 was higher in the upper part of the range values, reaching up to 258.80 pg/mL (excluding one extreme value of 1788.4 pg/mL), than in the lower part of the range values, where the minimum value was 0.14 pg/mL. Two other conventional markers of inflammation, NLR and CRP, also showed low variability and distributions being skewed around the median values.

It can be seen from correlation analyses (Table 2) that measures of the body’s shape and nutritional status (BMI, wc, and mac) are strongly correlated with each other. However, only two variables, total cholesterol, and LDL (Low Density Lipoprotein) cholesterol, indicating whether there is hypercholesterolemia or not, showed multicollinearity (Cholesterol: 9.33, LDL: 8.70), but these variables were not selected in predictive models (see Table 3).

We assessed which variables significantly changed among quartiles of IL-37 using the following cut-off values: between quartiles 1 and 2–3.40, between quartiles 2 and 3–13.40, and between quartiles 3 and 4–38.00. There were several variables that showed significant variations. These included erythrocytes, hemoglobin, and hematocrit, indicating chronic inflammation, and variables HbA1c (a borderline significance) and LDL-cholesterol, indicating metabolic disorders (Table S2—Supplementary Materials). All these variables can also be considered as signs of blood viscosity [52]. Of comorbid disorders, only CAD and gastrointestinal diseases were shown to be significant (Table S3—Supplementary Materials). In addition, the new generation of oral antidiabetic drugs, including DPP4inh (Dipeptidyl peptidase-4 inhibitors), SGLT2inh, and GLP1ra taken as a group, have shown significant variations.

Variables indicating the proportion of diabetic patients with CVD, including CAD and CHD, showed a tendency to increase according to the increasing quartile rank of IL-37. On the contrary, the variables hemoglobin, hematocrit, and HbA1c, and variables indicating the proportion of patients to whom new-fashioned oral antidiabetic drugs were prescribed, showed a decreasing tendency. It is worth mentioning here that chronic inflammation associated with metabolic conditions such as T2D may cause anemia (known as anemia of chronic disease). Anemia acts to decrease blood viscosity, while, on the contrary, the burden of inflammatory mediators and metabolic substances, such as glucose and lipids, tend to increase it. The net effect on blood viscosity of these two opposite tendencies may, in turn, affect the level of inflammation in a variable way. The group of variables—erythrocytes, hemoglobin, hematocrit, HbA1c, and LDL-cholesterol—all showing significant changes across quartiles of IL-37, but in a non-linear manner, are likely to reflect this scenario.

The degrees of renal function decline did not show variations according to increasing levels (expressed as quartiles) of IL-37 (Tables S2 and S3—Supplementary Materials).

As demonstrated by the regression models, many clinical characteristics of diabetic patients are associated with higher-ranked quartiles of IL-37 compared with the basic one showing both negative and positive associations (Table 3). In all models, the frailty index modulates associations of other variables with IL-37, negatively influencing IL-37 levels.

**Figure 3:**
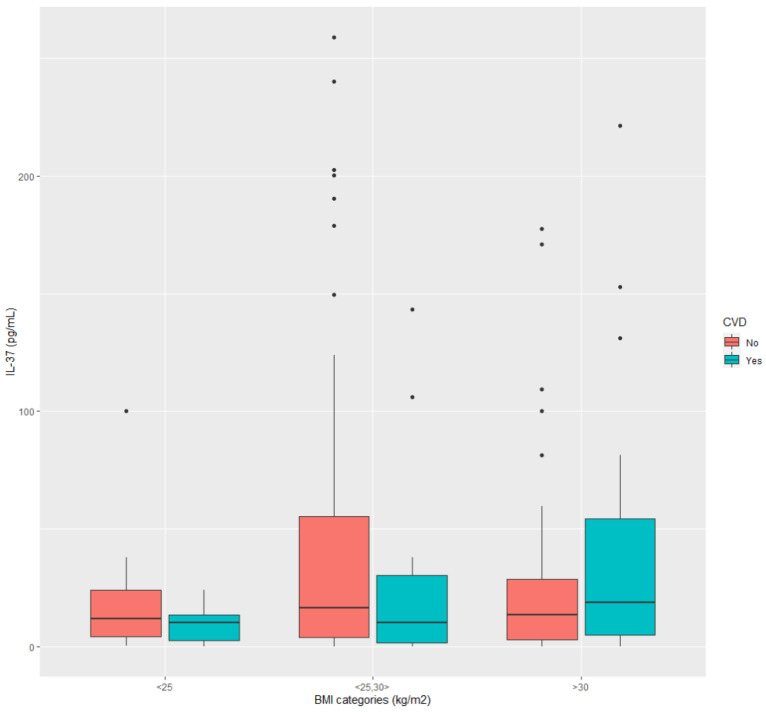
IL-37 in diabetic patients with/without CVD across categories of BMI: low (BMI < 25 kg/m^2^), normal (BMI 25–30 kg/m^2^) and high (BMI > 30 kg/m^2^).

**Figure 4:**
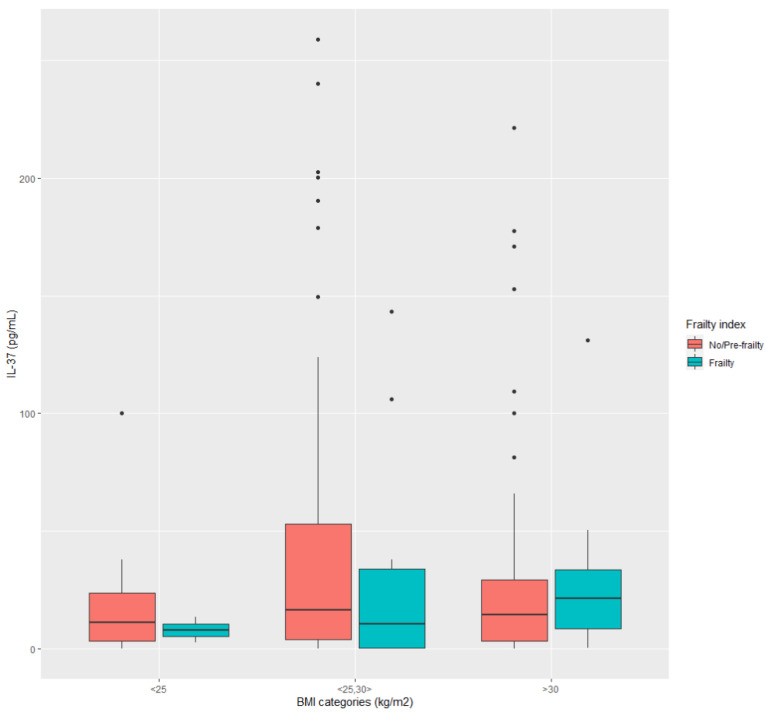
IL-37 according to frailty status (non frail + pre-frail/frail).

Figure 3 and Figure 4 show how IL-37 changes in dependence on two variables with opposite effects on IL-37 levels. The differences in both figures were statistically evaluated, i.e., if the respective data samples were normally distributed (Shapiro–Wilk test), the difference was investigated by the ANOVA test. Otherwise, we applied the Kruskal–Wallis test. The following were observed from Figure 3: in data sample BMI < 25 kg/m^2^ (no significant difference), BMI 25–30 kg/m^2^ (no significant difference), BMI > 30 kg/m^2^ (no significant difference), between BMI < 25 kg/m^2^ and BMI 25–30 kg/m^2^ (no significant difference), between BMI 25–30 kg/m^2^ and BMI > 30 kg/m^2^ (no significant difference). However, the tendency of elevated IL-37 in diabetic patients with CVD can be seen if they are obese (BMI > 30 kg/m^2^). Data from Figure 4 showed: in data sample BMI < 25 kg/m^2^ (no significant difference), BMI 25–30 kg/m^2^ (no significant difference), BMI > 30 kg/m^2^ (no significant difference), between BMI < 25 kg/m^2^ and BMI 25–30 kg/m^2^ (no significant difference), between BMI 25–30 kg/m^2^ and BMI > 30 kg/m^2^ (no significant difference). Similarly, as in the previous case, the tendency of IL-37 to increase can be seen in frail diabetic patients if they are obese, compared to those who are not.

The clinical utility of IL-37 cut-off values in identifying subgroups of diabetic patients, such as those with CAD, CHD, or other complications, does not seem promising (Table S4—Supplementary Materials). Only in a few cases were their use likely to be feasible (showing accuracy > 80%). These situations involve negative prediction (exclusion) of patients with low renal function (eGFR < 45 mL/min) or CAD, as well as positive prediction (recognition) of those with diabetic retinopathy or high-level comorbidity (>3 comorbid disorders). The levels of IL-37 that are higher than 38.2 pg/mL (from the upper quartile upward) are likely to indicate, with a high level of confidence, older diabetic patients with low BMI (<25 kg/m^2^).

**Figure 5:**
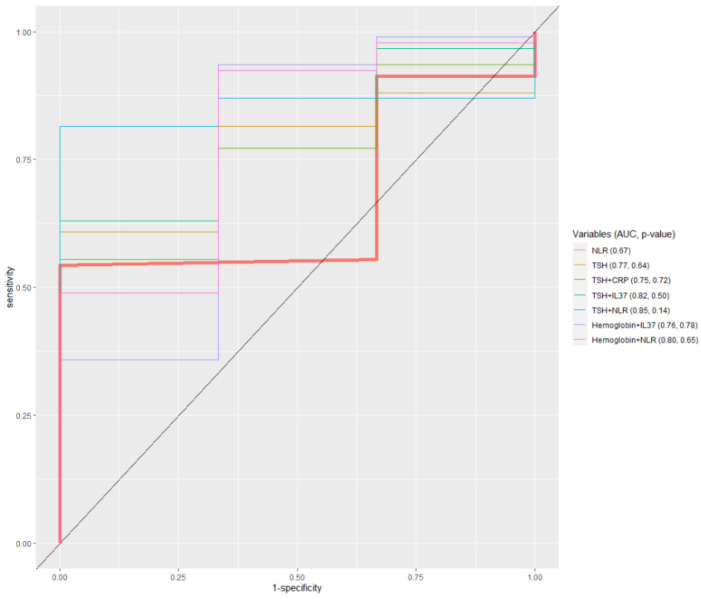
ROC model which discriminates between diabetic women with/without metabolic syndrome.

**Figure 6:**
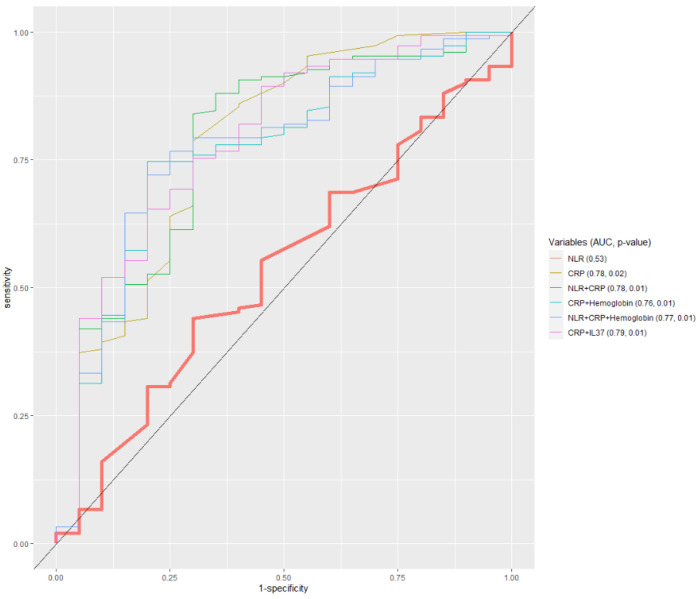
ROC model which discriminates between underweight diabetic patients (BMI < 25 kg/m^2^) and overweight/obese ones (BMI 25–30, and >30 kg/m^2^).

Interestingly, the only model that showed significant discriminative ability of determined cut-off values of IL-37 (expressed as AUC) was the one where IL-37 was combined with TSH in discriminating between women with and without MS (Table S5—Supplementary Materials) (Figure 5). The combination with TSH also improved the diagnostic accuracy of the basic NLR model. ROC analysis also indicates that models involving CRP are better than NLR in discriminating among diabetic patients according to BMI categories (Figure 6).

## 4. Discussion

In this sample of older diabetic patients, we have recorded significant variations in plasma levels of IL-37, ranging from 0.14 pg/mL to 258.80 pg/mL (except for one extreme value). In most participants, however, these values were at low levels, ranging from 3.40 to 38.00 pg/mL. According to the available evidence, these range values belong to the lower reference range for healthy adults [33]. There was a gentle rise in the 3rd, compared to the 2nd quartile, and a steep uprise in the upper quartile, so the distribution curve acquired the non-linear “J” shape. The low values of IL-37 in most patients may have been as a result of low inflammatory stimuli, intrinsically low anti-inflammatory response to inflammatory stimuli, or homeostasis breakdown due to organ damage. The fact that supports the first option is that most patients were metabolically well controlled, which may have attenuated the metabolic and inflammatory challenges. This statement is confirmed by HbA1c interquartile values ranging from 5.2% to 7.7%, which is within the recommended target values for older diabetic patients [39].

As indicated by the regression models, antidiabetic medications significantly associated with different levels of IL37 were metformin and DPP4 inh. These medications are known to have direct anti-inflammatory effects beyond the effect on lowering hyperglycemia, which can be due to their mechanisms of action [53]. Adverse associations of these medications with plasma IL-37 levels can be viewed in the light of evidence indicating obesity as a proinflammatory state and the fact that these medications are usually prescribed to obese diabetic patients as being weight-neutral [54]. The benefit of these medications may depend, as shown in this study (Figure 3), on whether obese diabetic patients have or do not have CVD. In the case of CVD, treating obese diabetic patients with these medications may suppress the protective homeostatic anti-inflammatory response. The need for personalization of diabetic therapy is especially emphasized when considering recent evidence that some IL-37 gene polymorphisms may be associated with low IL-37 production and increased susceptibility to T2D [21]. The contradictory results that hypolipidemic statin medications did not show associations with plasma IL-37 levels, despite their anti-inflammatory mechanism of action, also support this idea. More recently, statins’ alternating proinflammatory and anti-inflammatory effects have been observed, which are proposed to be clinical context-dependent or associated with gene polymorphisms [21,55]. To conclude, knowing more about the CV risk profile of patients in the sample is necessary to realize whether low IL-37 is prognostically beneficial or detrimental.

To make the range of IL-37 that we obtained in this study more reliable, we compared these values with the results of some prognostic studies from a similar setting. For example, even in the highest stage of hypertension, which is associated with overt atherosclerotic disease, plasma IL-37 levels reached values that were not higher than 50 pg/mL, which is consistent with the lower part of the upper IL-37 quartile in this study [30]. In patients with CHD, the plasma threshold of IL-37, shown to be prognostically negative, was 100 pg/mL, ranging within the limit of about 170 pg/mL [31]. These values apply to this study’s upper quartile range values of IL-37. In acute coronary syndrome, as an acute state of serious homeostasis breakdown, plasma IL-37 levels were shown to be lower than in the control and mostly below 40 pg/mL, which is consistent with the Q3 range values in this study [32].

To conclude, plasma IL-37 levels in these described pathologic conditions are consistent with Q3 and Q4 values of this study, where there was a noticeable rise in IL-37 away from the low values in Q1 and Q2 (above the cut-off of 3.40 pg/mL). However, this comparison still needs to answer whether patients in Q3 and Q4 are at increased CV risk. This uncertainty arises from the fact that the range values of the studies mentioned above are comparable with the reference range values for healthy adult controls, as reported in the recent meta-analysis [33]. Resolving this issue would require criteria reconsideration for subject selection in the control groups.

The large variability of circulating levels of IL-37, showing the skewed distribution we found in this study, is consistent with the view of T2D as a complex disease [35]. In order words, this implicates the existence of multiple patient subgroups. The complexity is especially emphasized in diabetic patients of older age for their increased risk for “harmful “geriatric conditions, such as sarcopenia, malnutrition, and frailty, that are known to change the course and clinical expression of T2D [56]. Of the utmost importance is recognizing frailty, because of its proven influence on negative outcomes and its modifying effect on treatments [57,58]. Frailty is a progressive disorder characterized by muscle loss, low activity, and disturbed homeostatic reserves in multiple organs and systems [59]. The closely related disorders, T2D, MS, and CVD, are all known to strongly associate with frailty [20,60,61,62]. In this study, about half of the patients were diagnosed with CVD, and a little fewer than half had pre-frailty/frailty. More than three-quarters of male and almost all female patients met the criteria for MS.

The results of this study reflect these associations. First, factors that were found to associate with IL-37 indicated metabolic disorders, chronic inflammation, and comorbidities of CVD. The second, pre-frailty/frailty status was highlighted as having a pivotal modifying role in these associations. Notably, a negative direction of associations of pre-frailty/frailty with IL-37, which was stably maintained across the regression models, is suggestive of the suppressive effect of pre-frailty/frailty on circulating levels of IL-37. Some clarifications come from the recent study, where it was found that plasma levels of IL-37 are lower in healthy elderly individuals than in middle-aged and young ones, despite elevations in proinflammatory markers [63]. These results suggest that IL-37 responsiveness to proinflammatory stimuli declines with age. Pre-frailty/frailty, rather than age per se, may have a role as the primary negative regulator of IL-37 [64], as our studies indicate that, at least in older diabetic patients, pre-frailty/frailty rates overcome those in the general population.

Moreover, associations between pre-frailty/frailty and IL-37 were shown to be non-linear, that is, discontinued among quartiles of IL-37. These results fit the evidence indicating that frailty status can change in severity (pre-frailty vs. frailty) with age and the number of comorbidities [65]. Lastly, these changes express as two metabolic phenotypes of frailty—one associated with obesity (myosteatosis) and another one associated with advanced stages of target organ damage, where frailty is characterized by both muscle and weight loss, and diffusely impaired physiological reserves (sarcopenic or cachectic frailty) [66,67,68]. Within this context, we can also consider the close associations between CVD and frailty. Evidence suggests that CVD can already appear in the early phases of T2D development, which is usually associated with obesity [69]. More often, and in more severe forms, CVD appears after a more extended period of T2D duration, which often co-exists with renal function decline; all these together increase the risk of sarcopenic frailty [34,70,71]. The role of non-cardiovascular comorbidities, which usually co-exist with T2D and CVD, in contributing to inflammation and the development of frailty, should also not be neglected. Literature evidence, together with results from this study, highlight the importance of musculoskeletal and gastrointestinal disorders in these conditions [72,73].

These results have pointed out one more fact. Abdominal obesity, presented with the variable wc, and its related disorders of MS, is, on the one hand, highly correlated with BMI (a sign of general obesity) and, on the other hand, with mac (a sign of muscle mass). In this regard, evidence indicated that associations of MS with frailty and mortality in the population of older adults remain stable across categories of BMI, including both obese and non-obese individuals with MS [74,75]. For example, a non-obese form of MS often accompanies chronic kidney disease (and is consistent with muscle mass loss and sarcopenic frailty) [76]. Taken together, if frailty is present in individuals with MS, this acts suppressively to IL-37; otherwise, obesity-related MS may regulate IL-37 through the intensity of inflammatory challenges [77]. Thus, as shown in Figure 4, to accurately validate circulating levels of IL-37 as a diagnostic tool in T2D patients, there is the need to recognize several MS phenotypes, relating them to the degree of frailty, sarcopenia, and categories of BMI.

When cut-offs of plasma IL-37 levels were considered with respect to their practical utility in identifying some specific subgroups of older diabetic patients, the best results were obtained in terms of negative prediction (exclusion) (NPV) of patients with low renal function (GFR < 45 mL/min) and those who have experienced CAD, and for positive prediction (recognition) (PPV) of those with low body mass (BMI < 25 kg/m^2^), those with diabetic retinopathy, and those who are characterized with comorbidities (>3). The problem with the practical usage of these results is in the fact that the estimated cut-off values belong to different parts of the IL-37 distribution curve (different quartiles) and that there is an overlap between the subgroups of diabetic patients when they are defined using one single label. For example, the subgroup with low body mass is suggestive of sarcopenic frailty. However, this can only be conclusively determined if we have insights into a more comprehensively defined profile of patients in this subgroup. Because of the complexity of older patients with T2D, using the circulating IL-37 levels as a diagnostic tool in patients with T2D will require different methodological approaches to those usually used. For example, in our recently published paper, we have suggested a profile-based approach [78]. In that study, we used the cytokine IL-17A, in addition to IL-37, as two major players of chronic inflammation and target organ damage in cardio-metabolic conditions, with the former having a pro-inflammatory role and the latter one acting as its suppressor. We have realized that these two cytokines are differently regulated, as indicated by their non-linear correlations, which may depend on the degree to which inflammation, tissue repair process, and fibrosis, take place in target organs. Many factors are likely to dictate the rates of these processes, which at least include patients’ age and gender, the time of T2D onset, being obese or not at the time of T2D onset, T2D and hypertension duration, and comorbidity-related frailty. Thus, neither IL-37 alone, or combinations of IL-17A and IL-37, can be used to predict T2D progression and bad outcomes, without knowing the wider contexts in which cytokine patterns are embedded. Moreover, variable clustering (based on their “natural” tendency to associate together), could be a way to indicate reliable patient subgroups (phenotypes) which differ from each other in risks for bad outcomes. As we have learned from previous experience, inflammatory markers are only moderate predictors in the setting of CVD, even if used in combination with each other or with other, organ-specific, and pathway-specific biomarkers [79,80]. In this study, the classical marker of inflammation, CRP, taken alone or in combination with other markers of inflammation, including IL-37, was indicated to be feasible in separating older diabetic patients according to whether they are overweight/obese or not. However, this finding is of little practical value because it remains unknown whether patients with low–normal BMI are relatively healthy diabetics or diabetics with sarcopenic frailty. Exciting results also indicate that TSH, used alone or in combination with markers of inflammation, is a potential biomarker for identifying older female diabetic patients with MS.

According to both our results (Table 3, the regression model “C”) and the literature evidence, the frailty status is able to modify correlations between TSH and IL-37. In our recently published review paper, we elaborated the role of TSH—a sign of the activated hypothalamus-pituitary-thyroid (HPT) axis—in conditions associated with inflammation and impaired glucose-related metabolism, such as aging and cardio-metabolic conditions [81]. In brief, a hypothyroid state in older individuals, indicated by increased TSH, is viewed as the system’s homeostatic reaction which takes place when IL-37, as a major tissue homeostatic mechanism under inflammatory conditions, fails to restore the metabolic costs of inflammation. This explains the negative correlation between TSH and IL-37 in the regression model. In this respect, in numerous observational studies, obesity and MS have been shown to strongly correlate with TSH. In the case of the system’s homeostasis failure, such as in fully developed frailty, the HPT axis also tends to be disrupted. Thus, TSH can be used as a biomarker of older diabetic women with MS if they are separated according to the frailty status (as non-frail, pre-frail, frail). It is worth mentioning in this part of the discussion that it is still unclear how many entities of MS female diabetic patients may involve, considering variations in factors such as BMI, stages of renal function decline, age, and the presence of CVD. Obviously, there is the need to change the research approaches in complex conditions such as T2D. We are now witnessing the new wave in research on T2D, which has become possible due to technological advances in molecular biology and methods for data analysis. The focus is on identifying metabolic phenotypes or pathways that can optimally support the personalized management of diabetic patients [82,83]. In this elaborative study, we have revealed limitations of using classical data analysis approaches to determine the diagnostic utility of the cytokine IL-37 in older patients with T2D, and we have laid a foundation for introducing new methodology approaches.

## 5. Conclusions

The results show that the regulation of circulating levels of IL-37 in older diabetic individuals is highly complex, mostly due to the high heterogeneity of these patients. Frailty was shown to have a suppressive effect on IL-37 circulating levels and a modifying role in associations of metabolic and inflammatory factors with IL-37, including the effect of treatments. Situations in which IL-37 attained clinically significant discriminating ability included the model of IL-37 and C-Reactive Protein in differentiating diabetic patients with low/normal–high BMI (<25/≥25 kg/m^2^), and the model of IL-37 and Thyroid Stimulating Hormone in discriminating between women with/without metabolic syndrome.

## Figures and Tables

**Table 1:** Markers of inflammation.

Markers of Inflammation	Median (IQR)	Mean (SD)
Total No. of Leukocytes (×10^9^/L)		7.58 (1.77)
No. of Lymphocytes (×10^3^/mL)	2.45 (1.17)	
No. of Neutrophils (×10^3^/mL)	3.99 (1.43)	
Lymphocytes %		34.26 (8.65)
Neutrophils %		53.06 (8.37)
NLR	1.60 (0.90)	
CRP (mg/L)	1.90 (2.20)	
Hb (g/L)	143.00 (18.00)	
IL-37 (pg/mL) *	13.40 (34.60)	

* One extreme value was excluded. NLR—neutrophil-to-lymphocyte ratio; CRP—C-reactive protein; Hb—hemoglobin; IL-17A—interleukin-17A.

**Table 2:** The result of correlation analyses.

Variable	Variable	Correlation Coefficient
Age	Hypertension duration	0.61
wc	mac	0.73
wc	BMI	0.78
Erythrocyte number	Hematocrit	0.83
Glucose	HbA1C	0.68
Cholesterol	LDL	0.91

**Table 3:** Multinomial logistic regression models for quartiles of IL-37. All models were adjusted for age, gender, T2D duration, and the frailty index. The tables visualized only variables marked as important by the respective regression model (*p*-value lower than 0.05).

(A) “Markers of inflammation” Model—variables in the input: erythrocyte number, leukocyte number, hemoglobin, hematocrit, neutrophils %, lymphocytes %, and CRP; AIC: 491.00						
	**Quartile 2**		**Quartile 3**		**Quartile 4**	
	**z-Value** **(*p*-Value)**	**OR (95% CI)**	**z-Value** **(*p*-Value)**	**OR (95% CI)**	**z-Value (*p*-Value)**	**OR (95% CI)**
Frailty index = 1	−2.42 (0.02)	0.24 (0.10–0.64)			−2.90 (0.003)	0.17 (0.06–0.46)
Frailty index = 2					−2.04 (0.04)	0.20 (0.05–0.73)
Erythrocyte					−3.22 (0.001)	0.02 (0.002–0.14)
CRP					2.44 (0.01)	1.35 (1.10–1.66)
Neutrophils %					2.03 (0.04)	1.12 (1.02–1.23)
(B) “Anthropometric measures” Model—variables in the input: BMI, wc, mac; AIC: 489.14.						
	**Quartile 2**		**Quartile 3**		**Quartile 4**	
	**z-Value ** **(*p*-Value)**	**OR (95% CI)**	**z-Value ** **(*p*-Value)**	**OR (95% CI)**	**z-Value ** **(*p*-Value)**	**OR (95% CI)**
Frailty index = 1	−2.16 (0.03)	0.30 (0.12–0.75)			−2.17 (0.03)	0.29 (0.11–0.74)
Wc			−2.70 (0.01)	0.92 (0.87–0.97)	−1.96 (0.04)	0.94 (0.90–0.99)
Mac			2.77 (0.01)	1.44 (1.16–1.79)	2.19 (0.03)	1.33 (1.07–1.64)
(C) “Laboratory tests” Model—variables in the input: eGFR, fasting glucose, HbA1c, triglycerides, total cholesterol, LDL-cholesterol, HDL-cholesterol, TSH, and uric acid; AIC: 506.91.						
	**Quartile 2**		**Quartile 3**		**Quartile 4**	
	**z-Value ** **(*p*-Value)**	**OR (95% CI)**	**z-Value ** **(*p*-Value)**	**OR (95% CI)**	**z-Value ** **(*p*-Value)**	**OR (95% CI)**
Frailty index = 1	−1.98 (0.04)	0.31 (0.12–0.82)			−1.97 (0.04)	0.31 (0.12–0.82)
TSH					−1.97 (0.04)	0.74 (0.58–0.95)
(D) “Cardio-metabolic comorbidities” Model—variables in the input: hypertension = no, <10 years of duration, >10 years of duration, CAD, CHD, peripheral artery dis., cerebrovascular dis., retinopathy, MS (M, F), smoking habit; AIC: 418.00.						
	**Quartile 2**		**Quartile 3**		**Quartile 4**	
	**z-Value ** **(*p*-Value)**	**OR (95% CI)**	**z-Value ** **(*p*-Value)**	**OR (95% CI)**	**z-Value ** **(*p*-Value)**	**OR (95% CI)**
Frailty index = 1	−2.25 (0.02)	0.25 (0.09–0.69)			−1.99 (0.04)	0.30 (0.11–0.81)
Frailty index = 2	−2.17 (0.03)	0.13 (0.03–0.61)				
CAD = 1			2.13 (0.03)	4.47 (1.40–14.25)		
(E) “Other comorbidities” Model—variables in the input: diagnosis of osteoporosis, osteoarthritis, low back pain, anxiety, COPD/asthma, gastrointestinal disorders, thyroid gland disorders, urogenital disease; AIC: 496.12.						
	**Quartile 2**		**Quartile 3**		**Quartile 4**	
	**z-Value ** **(p-Value)**	**OR (95% CI)**	**z-Value ** **(p-Value)**	**OR (95% CI)**	**z-Value ** **(p-Value)**	**OR (95% CI)**
Frailty index = 1	−2.69 (0.01)	0.19 (0.07–0.53)			−2.95 (0.003)	0.16 (0.06–0.45)
Low back pain = 1	−2.51 (0.01)	0.18 (0.06–0.56)	−2.35 (0.02)	0.21 (0.07–0.62)	−2.00 (0.04)	0.27 (0.09–0.79)
Gastro-intestinal dis = 1	2.37 (0.02)	4.59 (1.60–13.20)	2.36 (0.02)	4.25 (1.55–11.66)	2.52 (0.01)	4.90 (1.74–13.80)
(F) “Comorbidity level and functional disorders” Model—variables in the input: frailty index (0,1,2), nutritional status (categories), urinary incontinence, number of comorbidities, and number of medications prescribed; AIC: 489.07.						
	**Quartile 2**		**Quartile 3**		**Quartile 4**	
	**z-Value** **(*p*-Value)**	**OR (95% CI)**	**z-Value** **(*p*-Value)**	**OR (95% CI)**	**z-Value** **(*p*-Value)**	**OR (95% CI)**
Frailty index = 1	−2.65 (0.01)	0.21 (0.08–0.55)			−2.63 (0.01)	0.22 (0.08–0.57)
Frailty index = 2	−2.21 (0.03)	0.11 (0.02–0.57)				
Urinary incontinence = 1					2.01 (0.04)	5.35 (1.36–21.12)
(G) “Antidiabetic drugs” Model—variables in the input: metformin, sulfonylureas, pioglitazone, DPP4, GLP1ra, SGLT2-inh, insulin therapy; AIC: 503.36.						
	**Quartile 2**		**Quartile 3**		**Quartile 4**	
	**z-Value ** **(*p*-Value)**	**OR (95% CI)**	**z-Value ** **(*p*-Value)**	**OR (95% CI)**	**z-Value ** **(*p*-Value)**	**OR (95% CI)**
Frailty index = 1	−2.25 (0.02)	0.26 (0.10–0.70)			−2.04 (0.04)	0.29 (0.11–0.79)
Metformin = 1	−2.20 (0.03)	0.21 (0.06–0.67)				
DPP4 = 1					−2.63 (0.01)	0.16 (0.05–0.50)
(H) “Other medications” Model—variables in the input: NSAIL, ACE-INH or ARBs, calcium channel blockers, beta-blockers, diuretics, and statins. No one variable showed significance (all *p*-values higher than 0.05); AIC: 511.91						

## Data Availability

The data presented in this study are available on request from the corresponding author. The data are not publicly available due to unfinished doctoral work.

## Supplementary Materials

The following supporting information can be downloaded at: https://www.mdpi.com/article/10.3390/ijerph20043695/s1. Table S1. (a) Patient characteristics (numerical variables), (b) Patient characteristics (categorical variables); Table S2. Differences in distributions of examined variables among quartiles of IL-17A (numerical variables); Table S3. Differences in distributions of examined variables among quartiles of IL-17A (categorical variables); Table S4. Optimal cut-off values of IL-37 and other markers of inflammation for discriminating between subgroups of patients diagnosed with T2D with respect to the following measures: sensitivity, specificity, negative predictive value, and positive predictive value (ROC-analysis); Table S5. The area under ROC (AUC) for IL-37 and classical markers of inflammation: NLR, CRP and Hemoglobin, and combinations of markers. NLR is used as the basis for comparison.

## Abbreviations

ACE-INH: Angiotensin-converting enzyme inhibitors; AIC: Akaike Information Criterion; ANOVA: one-way analysis of variance; ARB: Angiotensin receptor blockers; AUC: the Area Under Curve; BMI: Body mass index; DNA: Deoxyribonucleic acid; CAD: Coronary artery disease; CHD: Chronic heart disease; CRP: C-reactive protein; CV: Cardiovascular; CVD: Cardiovascular disease; DPP4: Dipeptidyl Peptidase IV Inhibitors; eGFR: estimated glomerular filtration rate; GLP1: Glucagon-like peptide-1; Hb: Hemoglobin; HDL: High-density lipoprotein; IL-17A: interleukin-17A; IL-37: interleukin-37; IQR: interquartile range; MAC: mid-arm circumference; MDRD: Modification of Diet in Renal Disease; MLR: multinomial logistic regression; mRNA: Messenger RNA; MNA-SF: Mini Nutritional Assessment—Short Form; MS: Metabolic syndrome; NCP ATP III: National Cholesterol Education Program, Adult Treatment Panel III; NLR: neutrophil-to-lymphocyte ratio; NPV: negative predictive value; NSAID: non-steroidal anti-inflammatory drugs; PC: primary care; ROC: Receiver Operating Characteristics Curve; T2D: Diabetes Type 2; SGLT2: Sodium-glucose transport protein 2; TG: triglycerides; Treg: regulatory T cells; TSH: Thyroid stimulating hormone; VIF: Variance Inflation Factor; Wc: waist circumference
