# Supplementary material for: A Critical Appraisal of the Diagnostic and Prognostic Utility of the Anti-Inflammatory Marker IL-37 in a Clinical Setting: A Case Study of Patients with Diabetes Type 2
Source: Int J Environ Res Public Health. 2023 Feb 19;20(4):3695. doi: 10.3390/ijerph20043695 (PMC9966907; doi:10.3390/ijerph20043695)
Supplement: Supplementary file 1 [file ijerph-20-03695-s001.zip › Table S1.pdf]

Table S1a. Patient characteristics (numerical variables).

Variable	Median (IQR)	Mean (SD)
Age (years)	66.00 (12.00)	
Wc (cm)	103.00 (13.00)	
Mac (cm)	30.00 (4.00)	
BMI	29.72 (6.18)	
HbA1C (%)	6.90 (1.70)	
Diabetes duration (years)	8.00 (10.00)	
Hypertension duration (years)	10.00 (8.00)	
Erythrocyte number (x 10^{12}/L)		4.79 (0.44)
Haematocrit (%)	42.30 (4.70)	
Glucose (mmol/L)	7.95 (3.18)	
Total cholesterol (mmol/L)	5.20 (1.70)	
LDL (mmol/L)		3.21 (1.04)
HDL (mmol/L)	1.31 (0.40)	
Triglycerides (mmol/L)	1.73 (0.90)	
TSH (mIU/L)	2.52 (1.70)	
Uric acid (μmol/L)	320.00 (80.00)	

Wc = waist circumference; Mac = mid arm circumference; LDL = Low-density lipoprotein; HDL = High-density lipoprotein; TSH = thyroid-stimulating hormone

Table S1b. Patient characteristics (categorical variables).

Variable	Absolute No.
Age (years)	50-65 = 79<br>66-75 = 63<br>>75 = 28
Gender (M,F)	M = 44%
BMI (kg/m^2)	<25 = 20<br>25-30 = 67<br>>30 = 83
Nutritional status (MNA-test screening score)	at risk for malnutrition = 15.3%
Smoking habit	Never = 68<br>Current = 31<br>Ex = 71
Self-reported walking difficulties	Yes = 89.4%
frailty index (0 = robust; 1 = prefrail; 2 –frail)	0 = 97<br>1 = 42<br>2 = 31
Metabolic Syndrome<br>(M)<br>(F)	Yes = 32.9%<br>Yes = 54.1%
HbA1C (%)	<6.5 = 45<br>≥6.5 <7.5 = 60<br>≥7.5<8.5 = 32<br>≥8.5 = 33

eGFR (ml/min/1.73 m²)	<45 = 14<br><60 ≥45 = 39<br>≥60 <90 = 63<br>>90 = 54
Diabetes duration (years)	1-3y = 55<br>4-10y = 57<br>11-20y = 51<br>>20y = 7
Hypertension duration (years)	1-5y = 27<br>6-10y = 62<br>11-20y = 76<br>>20y = 5
No. of comorbidities	≤3 = 4.7%
Hypertension	Yes = 89.4%
One or more of: CAD, CHD, Cerebrovascular Dis., Periphery Artery Dis.	Yes = 51.2%
CAD (Coronary Artery Dis.)	Yes = 34.7%
CHD (Chronic Heart Dis.)	Yes = 48.8%
Diabetic retinopathy	Yes = 28.2%
Chronic respiratory disease (chronic obstructive pulmonary dis. or asthma)	Yes = 8.2%
Gastro-intestinal dis.	Yes = 42.4%
Osteoporosis	Yes = 45.9%
Osteoarthritis	Yes = 50.6%
Low back pain	Yes = 65.3%
Thyroid gland dis.	Yes = 19.4%
Urogenital dis.	Yes = 29.4%
Incontinentio urinae	Yes = 15.3%
Anxious disorders	Yes = 60.6%
Total No. of medications prescribed	≤3 = 5.3%
Metformin	Yes = 72.9%
Sulfonylureas	Yes = 22.4%
Pioglitazone	Yes = 5.9%
Metformin, Sulfonylureas, Pioglitazone = altogether (Old fashioned oral antidiabetics)	Yes = 84.70%
Dipeptidyl peptidase-4 inhibitor (DPP4)	Yes = 17.6%
Glucagon-like peptide-1 receptor agonist (GLP1)	Yes = 8.8%

Sodium-glucose co-transporter-2 (SGLT2)	Yes = 3.5%
DPP4, GLP1ra, SGLT2-inh = altogether (New fashioned oral antidiabetics)	Yes = 30.0%
Insulin therapy	Yes = 23.5%
ACE-INH or ARBs	Yes = 78.2%
Calcium channel blockers	Yes = 42.4%
Beta-blockers	Yes = 45.3%
Diuretics	Yes = 66.5%
Statins	Yes = 84.1%
NSAID	Yes = 71.2%

BMI = body mass index; MNA = test screening score = Mini Nutritional Assessment test screening; HbA1C = hemoglobinA1C; eGFR = estimated glomerular filtration rate; CVD = cardiovascular disease; CAD = coronary artery disease; CHD = chronic heart disease; DPP4inh = dipeptidyl peptidase-4 inhibitor; GLP1ra = glucagon-like peptide-1 receptor agonists; SGLT2inh = sodium-glucose cotransporter 2 inhibitors; ACE-INH = Angiotensin converting enzyme inhibitors; ARBs = Angiotensin receptor blockers; NSAID = Non-steroidal anti-inflammatory drugs.
