# Supplementary material for: A Critical Appraisal of the Diagnostic and Prognostic Utility of the Anti-Inflammatory Marker IL-37 in a Clinical Setting: A Case Study of Patients with Diabetes Type 2
Source: Int J Environ Res Public Health. 2023 Feb 19;20(4):3695. doi: 10.3390/ijerph20043695 (PMC9966907; doi:10.3390/ijerph20043695)
Supplement: Supplementary file 1 [file ijerph-20-03695-s001.zip › Table S2.pdf]

Table S2. Differences in distributions of examined variables among quartiles of IL-37. Numerical variables.

Variable	1st quartile 41 records	2nd quartile 41 records	3rd quartile 44 records	4th quartile 44 records	p-value	Post hoc test	
No. of lymphocytes	2.64 (0.90)*	2.56 (0.79)	3.30 (5.39)*	2.62 (1.06)*	0.88**		
No. of neutrophils	4.00 (0.74)	4.10 (1.32)	5.07 (6.70)*	4.23 (1.59)*	0.98**		
Lymphocyte %	33.80 (9.90)	34.20 (11.50)	33.20 (9.53)	32.90 (13.98)	0.74**		
Neutrophils%	52.80 (10.60)	53.20 (10.80)	53.50 (8.90)	54.50 (15.88)	0.79**		
NLR	1.59 (0.84)	1.51 (0.80)	1.83 (0.89)*	1.80 (0.79)*	0.73**		
CRP	2.18 (1.92)*	2.39 (1.93)*	3.14 (2.82)*	3.61 (3.65)*	0.38***		
eGFR	84.00 (43.00)	83.00 (43.00)	84.50 (35.00)	76.61 (25.09)*	0.39**		
Age	67.46 (9.62)*	64.95 (7.84)*	69.07 (8.83)*	67.07 (8.14)*	0.18***		
Waist circumference	106.00 (8.00)	100.00 (15.00)	103.34 (10.84)*	103.80 (12.10)*	0.44**		
mac	29.00 (3.00)	29.00 (4.00)	30.34 (2.88)*	30.30 (3.14)*	0.12**		
Diabetes duration	10.00 (12.00)	10.00 (11.00)	5.00 (9.75)	9.00 (7.25)	0.64**		
Hypertension duration	12.00 (7.00)	10.00 (6.00)	10.00 (8.00)	11.34 (5.20)*	0.28**		
BMI	29.21 (4.40)	28.83 (6.72)	*30.40 (3.92)	29.04 (7.97)	0.67**		
Total Leukocyte	7.58 (1.45)*	7.53 (1.55)*	7.38 (1.72)*	7.82 (2.26)*	0.77**		
Erythrocyte	4.48 (0.37)*	4.89 (0.43)*	4.81 (0.46)*	4.62 (0.46)*	**0.02*****	1-0	0.96****
						2-0	0.99
						3-0	0.08
						2-1	0.86
						**3-1**	**0.02***
						3-2	0.15
LDL	2.84 (1.08)*	3.45 (1.04)*	3.38 (1.04)*	3.18 (0.94)*	**0.04*****	**1-0**	**0.04******
						2-0	0.07
						3-0	0.43
						2-1	0.99
						3-1	0.62
						3-2	0.78
HDL	1.29 (0.35)	1.40 (0.50)	1.40 (0.49)	1.30 (0.30)	0.09**		
Hemoglobin	143.80 (10.68)*	148.00 (16.00)	140.20 (14.65)*	139.60 (12.86)*	**0.03****	1-0	1.00*****
						2-0	1.00
						3-0	0.65
						2-1	0.16
						**3-1**	**0.03***
						3-2	1.00
Hematocrit	42.64 (2.97)*	43.20 (3.90)	41.72 (4.17)*	41.49 (3.52)*	**0.04****	1-0	1.00*****
						2-0	1.00
						3-0	0.60
						2-1	0.25
						**3-1**	**0.04***
						3-2	1.00
Glucose	2.90 (3.60)	8.10 (3.00)	7.35 (2.05)	7.65 (2.83)	0.10**		
HbA1C	7.68 (1.28)*	6.90 (1.90)	6.80 (1.33)	6.70 (1.53)	**0.06****	1-0	1.00*****

						2-0	0.25
						3-0	0.19
						2-1	0.58
						**3-1**	0.45
						3-2	1.00
Triglycerides	1.60 (1.17)	1.77 (0.61)	1.83 (1.47)	1.77 (1.03)	0.32**		
Cholesterol	4.95 (1.38)*	5.57 (1.32)*	5.57 (1.42)*	5.00 (0.98)	0.15**		
TSH	2.80 (1.90)	2.35 (1.86)	3.03 (2.01)	2.41 (1.38)	0.29**		
Uric acid	315.00 (54.71)*	326.80 (62.84)*	322.50 (82.50)	330.00 (88.25)	0.40**		

Median (IQR)
*Mean (SD) – normally distributed
**Kruskal-Wallis rank sum test (the p-value is less than the significance level 0.05, we can conclude that there are significant differences between the groups)
***ANOVA test (p-value is less than the significance level 0.05, we can conclude that there are significant differences between the groups)
**** ANOVA post hoc test (Tukey's test) (significant difference with p-value less than 0.05)
***** KW post hoc test (Dunn's test with a Bonferroni correction) (significant difference with p-value less than 0.05)
Bolded – significant differences
