# Supplementary material for: A Critical Appraisal of the Diagnostic and Prognostic Utility of the Anti-Inflammatory Marker IL-37 in a Clinical Setting: A Case Study of Patients with Diabetes Type 2
Source: Int J Environ Res Public Health. 2023 Feb 19;20(4):3695. doi: 10.3390/ijerph20043695 (PMC9966907; doi:10.3390/ijerph20043695)
Supplement: Supplementary file 1 [file ijerph-20-03695-s001.zip › Table S3.pdf]

Table S3. Differences in distributions of examined variables among quartiles of IL-37. Categorical variables.

Variable	1st quartile 41 records	2nd quartile 41 records	3rd quartile 44 records	4th quartile 44 records	Total 170 records	p-value
eGFR						
<60	11 (26.8%)	13 (31.7%)	16 (39.0%)	13 (29.5%)	53 (31.2%)	0.78
60-90	14 (34.1%)	15 (36.6%)	14 (31.8%)	20 (45.5%)	63 (37.0%)	
>90)	16 (39.0%)	13 (31.7%)	14 (31.8%)	11 (25.0%)	54 (31.8%)	
frailty index						
0	17 (41.4%)	28 (68.3%)	22 (50.0%)	30 (68.2%)	97 (57.1%)	0.11
1	15 (36.6%)	8 (19.5%)	11 (25.0%)	8 (18.2%)	42 (24.7%)	
2	9 (22.0%)	5 (12.2%)	11 (25.0%)	6 (13.6%)	31 (18.2%)	
Gender = Male	21 (51.2%)	20 (48.9%)	15 (34.1%)	19 (43.2%)	74 (43.5%)	0.39
Age						
50-65	16 (39.0%)	23 (56.2%)	19 (43.2%)	21 (47.8%)	79 (46.5%)	0.46
66-75	18 (43.9%)	14 (34.1%)	14 (31.8%)	17 (38.6%)	63 (37.0%)	
>75	7 (17.1%)	4 (9.7%)	11 (25.0%)	6 (13.6%)	28 (16.5%)	
HbA1C						
<6.5	8 (19.5%)	9 (22.0%)	13 (29.5%)	15 (34.1%)	45 (26.5%)	0.08
≥6.5<7.5	12 (29.2%)	15 (36.6%)	17 (38.6%)	16 (36.4%)	60 (35.3%)	
≥7.5<8.5	9 (22.1%)	4 (9.7%)	9 (20.5%)	10 (22.7%)	32 (18.8%)	
≥8.5	12 (29.2%)	13 (31.7%)	5 (11.4%)	3 (6.8%)	33 (19.4%)	
Smoking						
Never	16 (39.0%)	14 (34.1%)	22 (50.0%)	16 (39.0%)	68 (40.0%)	0.19
Current + Ex	25 (61.0%)	27 (65.9%)	22 (50.0%)	28 (63.6%)	102 (60.0%)	
BMI						
<25	6 (14.6%)	6 (14.6%)	5 (11.4%)	3 (6.8%)	20 (11.8%)	0.69*
25-30	16 (39.0%)	16 (39.0%)	13 (29.5%)	22 (50.0%)	67 (39.4%)	
>30	19 (46.4%)	19 (46.4%)	26 (59.1%)	19 (43.2%)	83 (48.8%)	
Nutritional screening score =						0.57
normal nutritional state	32 (78.0%)	35 (85.4%)	39 (88.6%)	38 (86.4%)	144 (84.7%)	
NSAID = Yes	30 (73.2%)	28 (68.3%)	29 (65.9%)	34 (77.3%)	121 (71.2%)	0.65
Chronic obstructive pulmonary dis. Or asthma = Yes	6 (14.6%)	2 (4.9%)	3 (6.8%)	3 (6.8%)	14 (8.2%)	0.15*
CVD or Cerebrovascular D. = Yes	11 (26.8%)	9 (22.1%)	12 (27.3%)	10 (22.7%)	42 (24.7%)	0.92
CAD = Yes	7 (17.1%)	12 (29.2%)	18 (40.9%)	22 (50.0%)	59 (34.7%)	**< 0.01**
CHD = Yes	14 (34.1%)	1 (2.4%)	22 (50.0%)	27 (61.4%)	64 (37.6%)	0.08
Gastro-intestinal dis. = Yes	8 (19.5%)	19 (46.4%)	22 (50.0%)	23 (52.3%)	72 (42.4%)	**< 0.01**

Osteoporosis = Yes	5 (12.2%)	9 (22.0%)	11 (25.0%)	13 (29.5%)	38 (22.4%)	0.27
Osteoarthritis = Yes	17 (41.4%)	22 (53.7%)	21 (47.7%)	26 (59.1%)	86 (50.6%)	0.40
Low back pain = Yes	28 (68.3%)	25 (61.0%)	28 (63.6%)	30 (68.2%)	121 (71.2%)	0.87
Thyroid gland dis. = Yes	9 (22.0%)	9 (22.0%)	7 (15.9%)	8 (18.2%)	33 (19.4%)	0.87
Incontinentio urinae = Yes	3 (7.3%)	4 (9.7%)	11 (25.0%)	8 (18.2%)	26 (15.3%)	0.09
Urogenital dis = Yes	10 (24.4%)	12 (29.2%)	13 (29.5%)	15 (34.1%)	50 (29.4%)	0.81
Anxiety = Yes	18 (43.9%)	27 (65.9%)	30 (68.2%)	27 (61.4%)	102 (60.0%)	0.10
Diabetic retinopathy = Yes	12 (29.2%)	13 (31.7%)	14 (31.8%)	9 (20.5%)	48 (28.2%)	0.60
Metformin = Yes	32 (78.0%)	27 (65.9%)	31 (70.5%)	34 (77.3%)	124 (72.9%)	0.54
Sulfonylureas = Yes	9 (22.0%)	8 (19.5%)	9 (20.5%)	12 (27.3%)	38 (22.4%)	0.83
Pioglitazone = Yes	1 (2.4%)	3 (7.3%)	3 (6.8%)	3 (6.8%)	10 (5.9%)	0.32*
Old fashioned oral antidiabetics = Yes	34 (82.9%)	33 (80.5%)	37 (84.1%)	40 (90.9%)	144 (84.7%)	0.58
DPP4 = Yes	12 (29.2%)	8 (19.5%)	6 (13.6%)	4 (9.1%)	30 (17.6%)	0.09
SGLT2-inh = Yes	3 (7.3%)	1 (2.4%)	2 (4.5%)	0 (0.0%)	6 (3.5%)	0.24*
GLP1ra = Yes	6 (14.6%)	4 (9.7%)	3 (6.8%)	2 (4.5%)	15 (8.8%)	0.68*
New fashioned oral antidiabetics = Yes	21 (51.2%)	13 (31.7%)	11 (25.0%)	6 (13.6%)	51 (30.0%)	**< 0.01**
Insulin = Yes	10 (24.4%)	11 (26.8%)	11 (25.0%)	8 (18.2%)	40 (23.5%)	0.80
ACE-INH or ARBSs = Yes	32 (78.0%)	30 (73.2%)	37 (84.1%)	34 (77.3%)	133 (78.2%)	0.68
Calcium channel blockers = Yes	14 (34.1%)	18 (43.9%)	20 (45.5%)	20 (45.5%)	72 (42.4%)	0.68
Diuretics = Yes	26 (63.4%)	26 (63.4%)	31 (70.5%)	29 (65.9%)	112 (65.9%)	0.89
Beta-blockers = Yes	14 (34.1%)	18 (43.9%)	23 (52.3%)	21 (47.8%)	76 (44.7%)	0.39
Hyperlipidemia (statins therapy) = yes	35 (85.4%)	33 (80.5%)	36 (81.9%)	39 (88.6%)	141 (82.9%)	0.73
Number of comorbidities ≤3	38 (92.7%)	38 (92.7%)	44 (100%)	42 (95.5%)	162 (95.3%)	0.40*
Number of medications prescribed ≤3	39 (95.1%)	38 (92.7%)	42 (95.5%)	42 (95.5%)	161 (94.7%)	0.38*

Metabolic Syndrome (Male)	15 (36.6%)	13 (31.7%)	15 (34.1%)	13 (29.5%)	56 (32.9%)	0.66*
Metabolic Syndrome (Female)	19 (46.3%)	26 (63.4%)	21 (47.7%)	26 (59.1%)	92 (54.1%)	0.32*

Pearson's chi-squared test
*Fisher test
