# Supplementary material for: A Critical Appraisal of the Diagnostic and Prognostic Utility of the Anti-Inflammatory Marker IL-37 in a Clinical Setting: A Case Study of Patients with Diabetes Type 2
Source: Int J Environ Res Public Health. 2023 Feb 19;20(4):3695. doi: 10.3390/ijerph20043695 (PMC9966907; doi:10.3390/ijerph20043695)
Supplement: Supplementary file 1 [file ijerph-20-03695-s001.zip › Table S4.pdf]

Table S4. Optimal cut-off values of IL-37 and other markers of inflammation for discriminating between subgroups of patients diagnosed with T2D concerning measures: sensitivity, specificity, negative predictive value, and positive predictive value (ROC-analysis).

subgroups	NLR	CRP	Hb	IL-37
eGFR <60/≥60	Cutoff value: 2.4	5.2	130	7.2
	Sensitivity: 0.32	0.23	0.89	0.74*
	Specificity: 0.90	0.87	0.28	0.40
	NPV: 0.75	0.72	0.54	0.77
	PPV: 0.59	0.44	0.73	0.35
eGFR <45/≥45	Cutoff value: 1.5	2.1	134	13.2
	Sensitivity: 0.86	0.86	0.79	0.79
	Specificity: 0.45	0.57	0.64	0.47
	NPV: 0.97	0.98	0.21	0.96
	PPV: 0.12	0.15	0.96	0.12
Frailty index 1 or 2 / 0	3.09	1.2	135	3.4
	0.09	0.81	0.83	0.84
	0.97	0.37	0.41	0.34
	0.44	0.57	0.64	0.61
	0.82	0.63	0.65	0.63
Slownes (walking difficulties) yes/no	1.59	1.4	137	6
	0.63	0.68	0.71	0.72
	0.49	0.47	0.5	0.5
	0.82	0.30	0.33	0.34
	0.26	0.82	0.83	0.17
HbA1c ≤6.8/>6.8 (median value)	2.01	2.5	141	7.2
	0.29	0.49	0.61	0.73
	0.75	0.70	0.51	0.43
	0.49	0.55	0.68	0.64
	0.57	0.65	0.58	0.54
HbA1c<7/≥7 (cutoff indicating good glucose control according to the international guidelines)	1.59	2.5	140	7.2
	0.61	0.51	0.65	0.73
	0.54	0.70	0.48	0.46
	0.56	0.61	0.61	0.61
	0.59	0.60	0.49	0.60
BMI < 25 /≥25	1.5	1.2	150	38.2
	0.7	0.79	0.4	0.26
	0.44	0.7	0.72	0.95
	0.92	0.30	0.90	0.15
	0.14	0.95	0.16	0.95
BMI >30/≤30	1.2	0.9	148	0.8
	0.80	0.93	0.42	0.88
	0.28	0.22	0.64	0.17
	0.56	0.76	0.51	0.6
	0.54	0.53	0.55	0.5
Dg chronic heart disease/no	1.59	1.8	142	14.6
	0.65	0.54	0.68	0.58
	0.57	0.51	0.59	0.58
	0.65	0.5	0.62	0.6

	0.58	0.55	0.65	0.55
Dg coronary artery disease /no	Cutoff value: 1.89	1.3	142	10.2
	Sensitivity: 0.42	0.73	0.62	0.78
	Specificity: 0.72	0.37	0.59	0.49
	NPV: 0.7	0.72	0.45	0.81
	PPV: 0.45	0.38	0.74	0.45
Dg CVD (any of chronic heart disease, coronary artery disease, periphery artery disease, cerebrovascular disease)	1.24	6.2	142	9.4
	0.79	0.14	0.68	0.67
	0.33	0.95	0.55	0.47
	0.53	0.60	0.70	0.51
	0.62	0.67	0.52	0.63
Dg incontinentio or urogenital disease / no	1.29	2.3	129	10.2
	0.82	0.48	0.9	0.69
	0.37	0.66	0.18	0.45
	0.78	0.42	0.5	0.72
	0.43	0.71	0.66	0.42
Dg anxiety /no	1.9	4.8	137	5.2
	0.41	0.24	0.79	0.77
	0.81	0.9	0.43	0.43
	0.47	0.44	0.76	0.55
	0.76	0.78	0.48	0.67
Dg diabetic retinopathy/no	1.51	2.1	134	28.8
	0.65	0.49	0.81	0.36
	0.48	0.6	0.4	0.77
	0.78	0.32	0.45	0.31
	0.33	0.76	0.78	0.8
Diabetes duration >10 years /≤10 years	1.3	1.4	134	15.6
	0.74	0.69	0.82	0.58
	0.33	0.45	0.38	0.62
	0.71	0.43	0.52	0.42
	0.36	0.71	0.72	0.74
No. of comorbidities >3/≤3	1.74	2.2	144	4.2
	0.39	0.75	1	0.74
	0.88	0.56	0.53	0.63
	0.06	0.98	1	0.1
	0.98	0.08	0.13	0.98
Metabolic Syndrome (Male)	Cutoff value: 1.3	1.4	153	4.4
	Sensitivity: 0.84	0.77	0.28	0.76
	Specificity: 0.40	0.43	0.82	0.35
	NPV: 0.72	0.63	0.51	0.56
	PPV: 0.46	0.59	0.61	0.56
Metabolic Syndrome (Male)	**TSH**			
	Cutoff value: 2.17			
	Sensitivity: 0.77			
	Specificity: 0.42			
	NPV: 0.38			
	PPV: 0.80			

Metabolic Syndrome (Female)	1.3<br>0.83<br>0.35<br>0.78<br>0.43	1.3<br>0.83<br>0.35<br>0.78<br>0.43	145<br>0.52<br>0.61<br>0.43<br>0.69	16.4<br>0.53<br>0.53<br>0.67<br>0.39
Metabolic Syndrome (Female)	**TSH**<br>Cutoff value: 2.35<br>Sensitivity: 0.61<br>Specificity: 1.00<br>NPV: 0.08<br>PPV: 1.00			

*values>80% were considered important.
