# Supplementary material for: A Critical Appraisal of the Diagnostic and Prognostic Utility of the Anti-Inflammatory Marker IL-37 in a Clinical Setting: A Case Study of Patients with Diabetes Type 2
Source: Int J Environ Res Public Health. 2023 Feb 19;20(4):3695. doi: 10.3390/ijerph20043695 (PMC9966907; doi:10.3390/ijerph20043695)
Supplement: Supplementary file 1 [file ijerph-20-03695-s001.zip › Table S5.pdf]

Table S5. The area under ROC (AUC) for IL-37 and classical markers of inflammation: NLR, CRP and Haemoglobin, and combinations of markers. NLR is used as the basis for comparison.

Subgroups	Inflammatory markers and their combinations	AUC (NLR basic)	p-value according to the NLR
eGFR <45/≥45	IL-37	0.60	0.40
	**NLR**	**0.67**	
	Hb	0.72	0.68
	CRP	0.74	0.40
	CRP + Hb	0.75	0.52
	NLR + CRP	0.76	0.21
	NLR + Hb	0.78	0.29
	NLR + CRP + Hb	0.79	0.23
eGFR <60/≥60	Hb+IL-37	0.45	0.04
	CRP	0.49	0.11
	IL-37	0.54	0.36
	Hb	0.55	0.51
	CRP + Hb	0.55	0.51
	**NLR**	**0.60**	
	NLR + Hb	0.63	0.24
Frailty index 1 or 2 / 0	CRP	0.54	0.45
	NLR + Hb	0.54	0.41
	NLR + IL-37	0.56	0.63
	Hb + IL-37	0.57	0.70
	Hb	0.59	0.93
	**NLR**	**0.60**	
	IL-37	0.60	0.92
	CRP + Hb	0.61	0.76
	NLR + CRP + Hb	0.63	0.46
Walking difficulties	IL-37	0.54	0.63
	**NLR**	**0.61**	
	CRP	0.63	0.95
	Hb	0.74	0.39
	NLR+CRP+Hb	0.77	0.27
HbA1c <7/≥7	Hb	0.56	0.92
	**NLR**	**0.56**	
	NLR+IL-37	0.58	0.48
	CRP	0.58	0.84
	IL-37	0.58	0.78
	CRP+Hb	0.60	0.61
	Hb+NLR	0.60	0.60
	CRP+IL-37	0.60	0.58
BMI<30/≥30	IL-37	0.50	0.87
	**NLR**	**0.51**	
	Hb	0.53	0.81
	NLR+Hb	0.53	0.38
	Hb+IL-37	0.54	0.67
	NLR+CRP	0.56	0.24
	CRP+Hb	0.58	0.25
	CRP	0.58	0.23
BMI<25/≥25	Hb	0.49	0.69
	**NLR**	**0.53**	
	NLR+IL-37	0.58	0.50
	IL-37	0.58	0.63
	NLR+CRP	0.78	0.01

	CRP+Hb<br>NLR+CRP+Hb<br>CRP<br>CRP+IL-37	0.76<br>0.77<br>0.78<br>0.79	0.01<br>0.01<br>0.02<br>0.01
Dg chronic heart disease/no	CRP<br>Hb<br>IL-37<br>**NLR**<br>HB + IL-37<br>NLR + Hb	0.50<br>0.61<br>0.61<br>**0.62**<br>0.64<br>0.65	0.07<br>0.90<br>0.95<br><br>0.73<br>0.35
Dg coronary artery disease/no	CRP<br>**NLR**<br>Hb<br>CRP + Hb<br>NLR + Hb<br>IL-37<br>Hb + IL-37	0.52<br>**0.58**<br>0.60<br>0.60<br>0.63<br>0.66<br>0.66	0.32<br><br>0.85<br>0.83<br>0.28<br>0.23<br>0.24
Dg diabetic retinopathy/no	CRP<br>IL-37<br>NLR + IL-37<br>NLR + Hb<br>Hb<br>**NLR**	0.53<br>0.55<br>0.59<br>0.60<br>0.60<br>**0.60**	0.35<br>0.56<br>0.81<br>1.00<br>0.86
No. of comorbidities >3 / ≤3	**NLR**<br>CRP<br>NLR + CRP<br>CRP + IL-37<br>Hb	**0.58**<br>0.60<br>0.66<br>0.66<br>0.80	 0.85<br>0.22<br>0.24<br>**<0.01**
Metabolic syndrome / no	IL-37<br>Hb<br>TSH<br>CRP + TSH<br>IL-37 + TSH<br>Hb + IL-37<br>CRP + Hb<br>CRP<br>**NLR**<br>NLR + TSH<br>NLR + Hb	0.50<br>0.53<br>0.51<br>0.55<br>0.56<br>0.56<br>0.57<br>0.57<br>**0.58**<br>0.58<br>0.59	0.19<br>0.69<br>0.51<br>0.59<br>0.76<br>0.74<br>0.90<br>0.89<br><br>0.97<br>0.74
Metabolic syndrome (Females)	**NLR**<br>CRP<br>CRP+Hb<br>TSH+CRP<br>Hb+IL-37<br>NLR+Hb<br>TSH+IL-37<br>TSH+NLR	**0.67**<br>0.67<br>0.70<br>0.75<br>0.76<br>0.78<br>0.82<br>0.85	 0.99<br>0.95<br>0.72<br>0.86<br>0.65<br>0.50<br>0.14
